# T-Cell Mediated Immune Responses Induced in *ret* Transgenic Mouse Model of Malignant Melanoma

**DOI:** 10.3390/cancers4020490

**Published:** 2012-04-26

**Authors:** Oliver Abschuetz, Wolfram Osen, Kathrin Frank, Masashi Kato, Dirk Schadendorf, Viktor Umansky

**Affiliations:** 1 Skin Cancer Unit, German Cancer Research Center (DKFZ), Heidelberg and Department of Dermatology, Venereology and Allergology, University Medical Center Mannheim, Ruprecht-Karl University of Heidelberg, Mannheim , Heidelberg 69120, Germany; 2 Division of Translational Immunology, German Cancer Center, Heidelberg 69120, Germany; 3 Unit of Environmental Health Sciences, Department of Biomedical Sciences, College of Life and Health Sciences, Chubu University, Aichi 487-8501, Japan; 4 Department of Dermatology, University Hospital Essen, Essen 45122, Germany

**Keywords:** melanoma, transgenic mouse model, T cells, immunization, cytokines, melanoma associated antigens

## Abstract

Poor response of human malignant melanoma to currently available treatments requires a development of innovative therapeutic strategies. Their evaluation should be based on animal models that resemble human melanoma with respect to genetics, histopathology and clinical features. Here we used a transgenic mouse model of spontaneous skin melanoma, in which the *ret* transgene is expressed in melanocytes under the control of metallothionein-I promoter. After a short latency, around 25% mice develop macroscopic skin melanoma metastasizing to lymph nodes, bone marrow, lungs and brain, whereas other transgenic mice showed only metastatic lesions without visible skin tumors. We found that tumor lesions expressed melanoma associated antigens (MAA) tyrosinase, tyrosinase related protein (TRP)-1, TRP-2 and gp100, which could be applied as targets for the immunotherapy. Upon peptide vaccination, *ret* transgenic mice without macroscopic melanomas were able to generate T cell responses not only against a strong model antigen ovalbumin but also against typical MAA TRP-2. Although mice bearing macroscopic primary tumors could also display an antigen-specific T cell reactivity, it was significantly down-regulated as compared to tumor-free transgenic mice or non-transgenic littermates. We suggest that *ret* transgenic mice could be used as a pre-clinical model for the evaluation of novel strategies of melanoma immunotherapy.

## 1. Introduction

Malignant skin melanoma is characterized by high metastatic potential and poor response to currently applied treatment modalities (like chemo- or radiotherapy), resulting in a very bad prognosis for patients with the advanced disease [1]. Moreover, the incidence of malignant melanoma is continuously rising worldwide [2] that requires the development of new alternative strategies for therapy of this aggressive disease. Well-documented melanoma immunogenicity has made different immunotherapies particularly attractive. Numerous evidences suggest that melanoma is an immunogenic tumor. For example, an infiltration of human melanoma lesions with T lymphocytes has been shown to correlate with the better clinical outcome [3]. Moreover, some patients displayed spontaneous histological tumor regression [4]. Large numbers of melanoma-associated antigens (MAA) have been identified as well as the development of spontaneous T cell reactions and antibody production against various antigens (such as Melan-A, gp100, tyrosinase or NY-ESO-1) has been reported in patients with advanced melanoma [5,6]. Adoptive transfer of autologous tumor antigen-specific CD8^+^ cytotoxic T cells (CTLs) has been demonstrated to improve the clinical outcome of stage IV melanoma patients [7,8,9].

Since CTLs are considered as effector cells mediating anti-tumor reactivity, numerous CTL epitopes derived from human and murine tumor-associated antigens have been described that could be used for the induction and characterization of tumor-reactive CTL responses [10,11]. In the transplantable B16 mouse melanoma model, specific CTL responses against the model MAA tyrosinase related protein (TRP)-2 have been extensively studied. In fact, an induction of TRP-2-specific CTL reactions has been shown to inhibit the formation of melanoma lung metastases [12,13]. Furthermore, TRP-2-specific immunization of C57BL/6 mice with established B16 melanomas resulted in the tumor eradication that was dependent on the antigen-specific CTL reactivity [14].

However, this tumor model is based on the transplantation of tumor cells, in which the tumor initiation and tumor-stroma interactions are not comparable with the clinical situation. In contrast to transplantation models, a *ret* transgenic mouse model closely resembles human melanoma regarding tumor genetics, histopathology and clinical development [15]. Mice expressing the human *ret* transgene in melanocytes controlled by the mouse metallothionein-I promoter-enhancer develop spontaneously malignant cutaneous melanoma lesions metastasizing to lymph nodes (LNs), lungs, the liver, brain, and bone marrow [15,16,17]. This metastatic profile is similar to that observed in melanoma patients [18]. Importantly, the C57BL/6 genetic background of *ret* transgenic mice allows investigation of H2^b^-restricted T-cell responses that are specific for described CD8 T-cell epitopes derived from MAA such as TRP-2.

The study of anti-tumor T-cell mediated immune reactions and the development of new immunotherapeutic strategies based on *ret* transgenic model requires a functionally intact T-cell compartment. The development of spontaneous anti-tumor immune responses mediated by CD8^+^ T cells and an accumulation of MAA-specific T cells in melanoma lesions of transgenic mice has been previously described by us and others [16,17,19,20,21]. Moreover, these CD8^+^ T cells were demonstrated to control metastatic progression since their depletion significantly accelerated visceral tumor outgrowth and reduced mouse survival [19,20]. Here we investigated antigen-specific and -unspecific T-cell-mediated immune responses in *ret* transgenic mice with or without macroscopic skin tumors. In comparison to non-transgenic C57BL/6 littermates, T cells from transgenic mice have been found to display a similar level of reactivity upon antigen-unspecific activation with anti-CD3 and anti-CD28 monoclonal antibodies (mAbs) or with a peptide derived from the strong model antigen ovalbumin (OVA). The same results have been also demonstrated upon stimulation with a peptide derived from the typical MAA TRP-2 that has been shown to be strongly expressed both in primary skin tumors and metastatic lesions in LNs, lungs and liver. We demonstrate that *ret* transgenic mice display no signs of reduction of T-cell reactivity and suggest that it could be used for the study of novel T-cell based melanoma immunotherapies.

## 2. Results

### 2.1. Expression of Melanoma Associated Antigens in Tumors of ret Transgenic Mice

**Figure 1:**
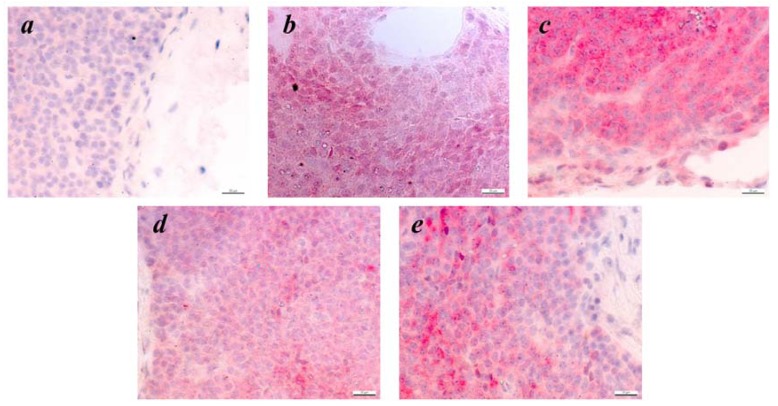
Expression of differentiation MAA in skin tumors of *ret* transgenic mice. Consecutive paraffin sections of mouse tumors (n = 20) were incubated with secondary Abs only (negative control, **a**) or stained with rabbit anti-mousepolyclonal Abs against TRP-2 (**b**), gp100 (**c**), TRP-1 (**d**) or tyrosinase (**e**). Finally, sections were counterstained with haemalaun. Original magnification, ×400.

**Figure 2:**
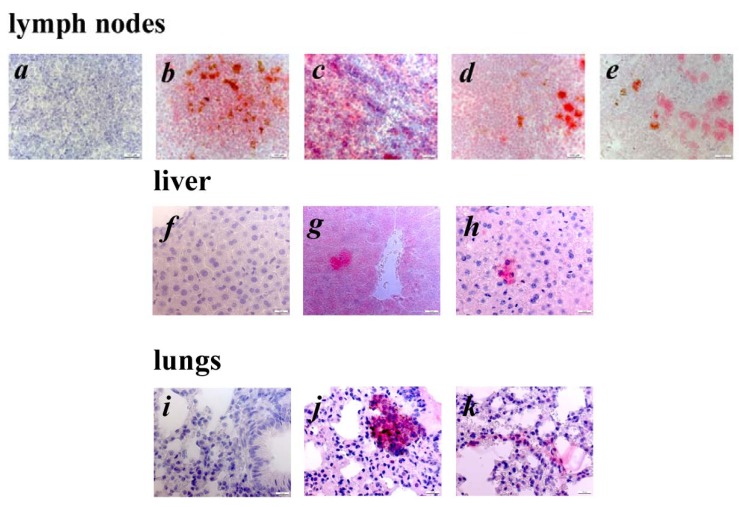
Detection of metastatic melanoma cells in distant organs of tumor bearing *ret* transgenic mice. Consecutive paraffin sections of LN (**a–e**), liver (**f–h**) and lungs (**i–k**) from tumor bearing *ret* transgenic mice (n = 10) were stained with rabbit anti-mouse polyclonal Abs against TRP-2 (**b**, **g**, **j**), TRP-1 (**c**), tyrosinase (**d**) and gp100 (**e**, **h**, **k**) and counterstained with hemalaun. Consecutive paraffin sections of LN (**a**), liver (**f**) and lungs (**i**) from non-transgenic littermates were used as control. Original magnification, ×400.

In this study, we used transgenic mice overexpressing the human oncogene *ret*. around 25% of all transgenic mice develop clinically visible cutaneous tumors on the face, back or on the tail. Other transgenic mice developed only microscopic skin tumors. All transgenic mice developed metastases in LNs and some distant organs like lungs or liver. Immunohistologic analysis of primary tumors using polyclonal Abs against well-known MAA tyrosinase, gp-100, TRP-1 and TRP-2 revealed the expression of these antigens in studied samples (Figure 1). Interestingly, both TRP-1 and TRP-2 showed a homogenous staining pattern (Figure 1b,d), whereas the expression of gp100 and tyrosinase was restricted to certain tumor cell clusters (Figure 1c,e). Remarkably, the expression of TRP-2 was detected in all skin tumors tested. Investigating metastatic lesions from LNs, liver and lungs, we also found the expression of TRP-2 (Figure 2b,g,j) and gp100 (Figure 2e,h,k). Furthermore, metastatic cells in LNs were demonstrated to be positive for TRP-1 and tyrosinase (Figure 2c,d). Taken together, the analysis of morphology and expression of typical antigenic markers suggest that the developed lesions are melanoma.

### 2.2. Antigen-Unspecific T-cell Reactions in ret Transgenic Mice

**Figure 3:**
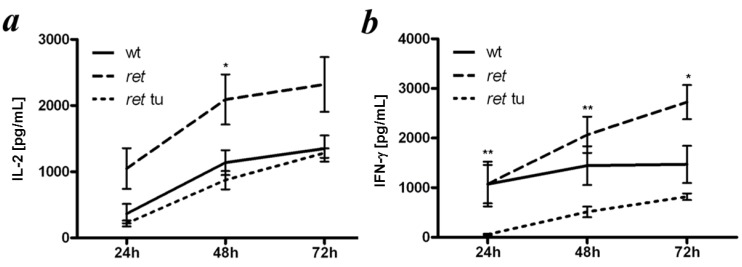
Reactivity of spleen T cells from *ret* transgenic mice upon antigen-unspecific stimulation. Splenocytes from tumor free (*ret* ) or tumor bearing transgenic mice (*ret* tu) or non-transgenic littermates (wt) were incubated overnight with anti-CD3 and anti-CD28 mAbs. The amount of secreted IL-2 (**a**) and IFN-γ (**b**) was determined by ELISA. Data (mean ± SEM of 4–5 mice per group) are expressed as pg/mL. * *p* < 0.05, *ret versus* wt or *ret versus ret* tu groups. ** *p* < 0.05, *ret versus ret* tu groups.

We next investigated the responses of T cells from *ret* transgenic mice induced by an antigen-unspecific stimulations with mAbs against CD3- and CD28 followed by measuring the production of IL-2 and IFN-γ. Spleen cells from *ret* transgenic mice without macroscopic tumors secreted similar amounts of IL-2 as compared to non-transgenic littermates (wild type mice; Figure 3a). Interestingly, splenocytes from tumor bearing mice showed less capacity to produce IL-2 (Figure 3a).

Studying the IFN-γ secretion at same conditions, we also found that splenocytes from tumor-bearing transgenic mice produced much less cytokine than cells from wild type mice or transgenic animals without macroscopic skin tumors (Figure 3b). Therefore, development of macroscopic cutaneous melanomas could impair the secretion of IL-2 and IFN-γ by T cells upon antigen-unspecific stimulation, whereas cells from transgenic tumor free mice were able to mount strong cytokine responses.

### 2.3. Analysis of CTL Responses against the Model Antigen OVA in ret Transgenic Mice

**Figure 4:**
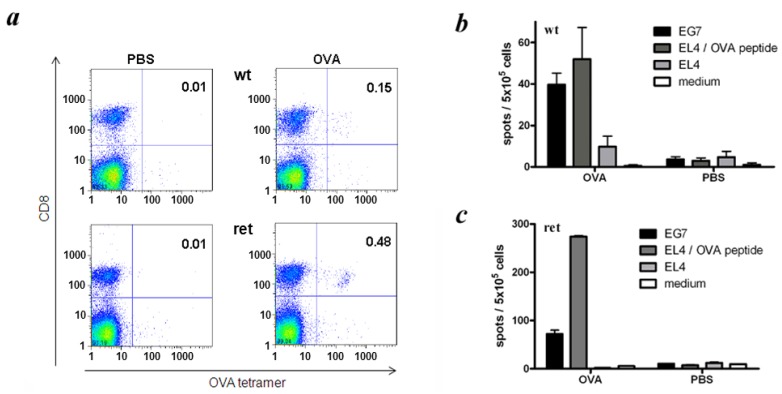
Induction of OVA-specific CD8^+^ T cell responses in *ret* transgenic mice. Tumor free *ret* transgenic mice (ret) or non-transgenic littermates (wt) were immunized with expression plasmid pcDNA3.1-OVA. Control mice were injected with PBS. Frequencies and functional activities of OVA-specific spleen CD8^+^ T cells were analyzed *ex vivo* by tetramer staining (**a**) and IFN-γ ELISPOT assays (**b**, **c**); (**a**). Representative dot plots for tetramer staining are shown; (**b**, **c**). Cumulative data (mean ± SEM; 9–11 mice per group) for IFN-γ secreting CD8^+^ T cells are presented as numbers of IFN-γ spots among 5 × 10^5 ^splenocytes.

Next we investigated the induction of antigen-specific T cell responses *in vivo* focusing on the strong model antigen OVA. Transgenic mice without visible tumors and non-transgenic littermates were immunized with OVA-encoding expression plasmids followed by the measurement of OVA-specific CTLs in spleens of vaccinated mice by tetramer staining. In addition, OVA-specific CTL reactivity was tested by IFN-γ ELISPOT assay performed directly *ex vivo*. As shown in Figure 4a, the frequency of OVA-specific CTLs among total spleen cells was found to be 0.15% upon DNA immunization of wild type C57BL/6 mice, whereas in *ret* transgenic mice, this value reached 0.48% at the same conditions.

The IFN-γ ELISPOT assay revealed that the reactivity of splenocytes from *ret* transgenic mice against OVA-expressing EG7 cells was significantly elevated as compared to cells from wild type mice (72 ± 8 and 40 ± 6 IFN-γ-producing cells per 5 × 10^5^ splenocytes respectively; *p* < 0.05; Figure 4b,c). Interestingly, when using EL4 cells pulsed with OVA-specific SIINFEKL peptide as target cells, the reactivity of splenocytes from transgenic mice was even higher (274 ± 2 and 52 ± 14 IFN-γ-secreting cells per 5 × 10^5^ splenocytes respectively; Figure 4b,c). These data are in accordance with the above mentioned higher frequency of OVA-specific CTL in transgenic mice upon the OVA immunization detected by tetramer stainings.

### 2.4. CTLs from Transgenic Mice Exerted MAA-Specific Reactivity upon Immunization

**Figure 5:**
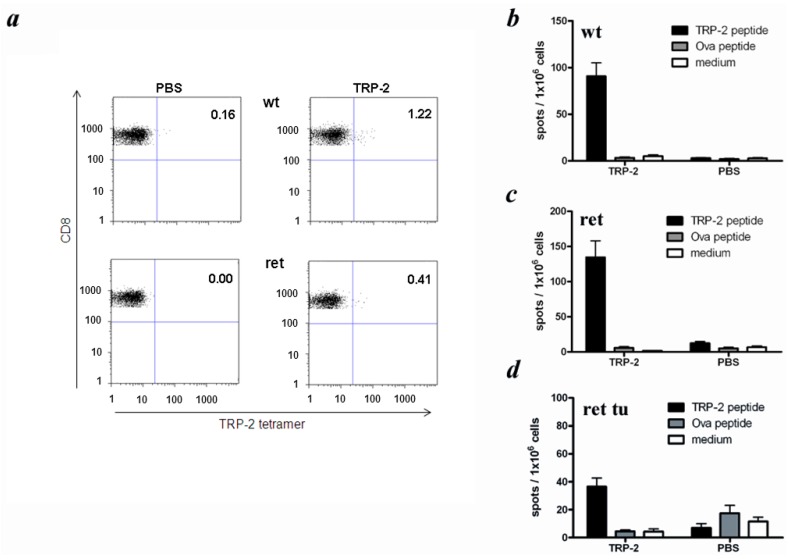
Induction of TRP-2-specific CD8^+^ T cell responses in *ret* transgenic mice. Mice with (ret tu) or without macroscopic tumors (ret) or non-transgenic littermates (wt) were immunized with the peptide derived from TRP-2. Frequencies and functional activities of TRP-2-specific spleen CD8^+^ T cells were analyzed *ex vivo* by tetramer staining (**a**) and IFN-γ ELISPOT assay (**b–d**). Control animals were injected with PBS. (**a**) Representative dot plots for tetramer staining are shown; (**b–d**) Cumulative data (mean ± SEM; 6–8 mice per group) for IFN-γ secreting CD8^+^ T cells are shown as numbers of IFN-γ spots among 1 × 10^6^ splenocytes.

Next, we studied CTL responses against the MAA TRP-2, which represents a well characterized differentiation antigen harboring a K^b^-restricted CTL epitope [22,23]. Since *ret* transgenic mice and C57BL/6 wild type mice share the same H2 haplotype (H2^b^), their TRP-2-specific CTL responses could be studied upon the immunization *in vivo* with the TRP-2-derived peptide TRP-2_180–188_. In wild type mice, the frequencies of tetramer-positive CTLs within total mononuclear spleen cells ranged from 0.3% to 1.2% upon immunization with the TRP-2 peptide, whereas spleens of immunized *ret* transgenic mice without macroscopic skin tumors contained from 0.3% to 0.4% TRP-2-reactive T cells (Figure 5a).

Testing in the ELISPOT assay the ability of spleen T cells to produce IFN-γ upon the TRP-2 peptide vaccination, we found that both wild type and tumor free transgenic animals displayed comparable amounts of IFN-γ-secreting T cells (Figure 5b,c), which was much lower than those in the same mouse groups after the immunization with OVA peptide (Figure 4b,c). Similar to the situation with the antigen-unspecific stimulation of spleen T cells *in vitro*, the TRP-2-specific reactivity of CD8^+^ T cells (reflected by the numbers of IFN-γ-secreting T cells upon the peptide immunization) from transgenic mice with clinically visible tumors was considerably lower than in both above mentioned groups (*p* < 0.05; Figure 5d). As expected, no reactivity was observed when the medium alone or unrelated peptide was used for *in vitro* re-stimulation. In addition, splenocytes of control, non-immunized mice showed no reactivity against any peptide after re-stimulation *in vitro* (Figure 5b–d).

In conclusion, *ret* transgenic mice exert unimpaired T cell-mediated immune responses upon antigen-unspecific and antigen-specific stimulation including the vaccination with peptide derived from typical MAA TRP-2.

## 3. Discussion

Development of innovative immunotherapeutic strategies for malignant melanoma depends on the availability of reliable animal models that display a similarity to the situation in melanoma patients. Most immunological animal studies on malignant melanoma have been performed so far using the transplantable B16 melanoma model [24,25]. While these melanoma cells express antigens showing a high homology to various human MAA, the model in general does not reflect human melanoma with regards to the initiation, genetic impairments, clinical development and histopathology. In this respect, recently developed autochthonous melanoma models seem to be more reliable [26,27,28]. We have chosen the *ret* transgenic mouse model, due to its close analogy to human melanoma regarding genetic alterations, pathology and tumor progression [15,16,17]. Importantly, primary skin tumors developed in *ret* transgenic mice were found to express several differentiation MAA such as TRP-1, TRP-2, gp100 and tyrosinase, which have been previously identified as targets for tumor-reactive T cells in melanoma patients [29,30]. Moreover, we demonstrated the expression of these MAA also in tumor cells infiltrating LNs, lung and liver. Such metastatic pattern is known to be typical for human malignant melanoma [18,31].

Since *ret* transgenic mice display H2^b^ haplotype, it was possible to analyze H2^b^-restricted CD8^+^ T cell responses specific for a strong model antigen OVA and for a typical MAA such as TRP-2 [23]. In our experiments, T cell-mediated immune reactions to antigen-unspecific stimulation *in vitro* (with anti-CD3 and anti-CD28 mAbs) and to antigen-specific activation *in vivo* (upon OVA-vaccination) in *ret* transgenic mice without macroscopic tumors appeared to be even more efficient than those in non-transgenic littermates. Interestingly, in transgenic mice without tumors immunized with the OVA-encoding DNA, the ELISPOT assay showed much higher T-cell reactivity against target cells loaded with OVA-derived peptide SIINFEKL than that in wild type mice. However, when OVA-transfected cells (EG7) with an endogenous antigen processing were used as targets, the difference in amounts of IFN-γ producing T-cells in these mouse groups was significantly lower suggesting a strong induction of CD8^+^ T cells with low affinity that are unable to recognize an antigen on EG7 transfectants but still can react against targets loaded with cognate peptides. Mechanisms of significantly higher activating capacity of T cells from *ret* transgenic mice upon OVA-vaccination are currently under investigation. Importantly, immunization with the peptide derived from the typical MAA TRP-2 induced much lower frequencies of TRP-2-specific CD8^+^ T cells in both *ret* transgenic and wild type mice as compared to amounts of OVA-specific CD8^+^ T cells detected after the respective vaccination. This might be explained by a higher immunogenicity of the xenogeneic model antigen OVA than that of the self-antigen TRP-2. Indeed, it has been reported that successful induction of TRP-2-specific CTLs in C57BL/6 mice upon DNA immunization was dependent on the presence of an IA^b^-restricted T helper epitope provided by the primary sequence of human (xenogeneic) TRP-2, but was absent in the murine TRP-2 [32]. When comparing *ret* transgenic mice with non-transgenic littermates, the capability to induce TRP-2-specific CD8^+^ T cell responses upon peptide immunization (measured by tetramer stainings and IFN-γ ELISPOT analysis) was found to be at the similar level.

It is important to note that in *ret* transgenic mice bearing macroscopic tumors, the T cell reactions induced by both antigen-unspecific stimulation *in vitro* and antigen-specific specific immunization *in vivo* were clearly impaired indicating a severe immunosuppression developing during melanoma progression. This immunosuppression was thought to be induced by the chronic inflammation characterized by the accumulation of inflammatory mediators (cytokines, chemokines, growth factors, reactive oxygen and nitrogen species, prostaglandins) as well as immunosuppressive leucocytes like CD11b^+^Gr1^+^ myeloid-derived suppressor cells (MDSCs), CD4^+^CD25^+^Foxp3^+^ regulatory T cells, M2 subset of macrophages, *etc*. in tumor bearing hosts [33,34,35,36]. Indeed, we have recently demonstrated that *ret* transgenic mice with clinically visible tumors contained an enhanced concentration of various chronic inflammatory factors including vascular endothelial growth factor (VEGF), transforming growth factor (TGF)-β, interleukin (IL)-1β, IL-6, GM-CSF, and IFN-γ as well as immunosuppressive leucocytes such as MDSCs and Tregs in the tumor microenvironment and peripheral lymphoid organs [37,38,39,40]. It is conceivable that this immunosuppressive network might affect effector CD8^+^ T cells also in the periphery. Furthermore, it has recently been reported that T cells could even promote melanoma progression in this model by favoring pro-tumoral properties of tumor infiltrating myeloid cells that further inhibit functions of immune effector cells [41].

Taken together, our study revealed that *ret* transgenic mice contain a functional T cell compartment capable of mounting specific T cell responses against not only a strong model antigen OVA but also against the typical MAA TRP-2 upon *in vivo* vaccination. Therefore, this autochthonous mouse melanoma model allows an investigation of the interactions between tumor and T cells at different phases of spontaneous melanoma progression. Furthermore, the development of the immunosuppressive network induced by chronic inflammation as well as its effects on the functional activity of endogenous and adoptively transferred melanoma-reactive T cells could be studied. We suggest that this model may be used for the development of novel strategies of human melanoma immunotherapy that include the neutralization of the immunosuppressive tumor microenvironment in combination with tumor vaccination or adoptive transfer of MAA-specific T cells.

## 4. Experimental Section

### 4.1. Mice

Transgenic mice expressing the human *ret* gene under the control of the murine metallothionein-I promoter on a C57BL/6 background (H2^b^) were kindly provided by I. Nakashima (Nagoya, Japan). Transgene expression was detected by RT-PCR with RNA from tail biopsies using following primers: 5'-AAAATGCAGTCAGATATGGA-3' and 5'-ACTCGGGGAGGGGTTC-3' respectively. As β-actin-specific primers, we used 5'-CACCGGAGAATGGGAAGCCGAA-3' and 5'-TCCACACAGATGGAGCGTCCAG-3'. Mice were crossed and kept under specific pathogen-free conditions in the animal facility of German Cancer Research Center. Spontaneous tumor development was assessed macroscopically twice per week. Experiments were performed in accordance with government and institute guidelines and regulations.

### 4.2. Unspecific Stimulation of T cells *in Vitro*

Splenocytes (10^6^ cells) depleted from erythrocytes by the ammonium chloride buffer were cultured in 24-well plates (TPP, Trasadingen, Switzerland) with soluble anti-CD3 and anti-CD28 mAbs (0.5 µg/mL each; BD Biosciences, Heidelberg, Germany). After 24, 48, and 72 h, cultures were harvested and cytokine concentrations in the supernatants were determined by ELISA.

### 4.3. Immunization of Mice

Peptide immunizations were performed as described elsewhere [42]. For DNA vaccination, mice were injected with pcDNA-OVA encoding chicken OVA (kindly provided by L. Gissmann, Heidelberg, Germany) as previously described [43].

### 4.4. Tetramer Staining and Flow Cytometry

Mouse APC-conjugated tetramers containing K^b^ and peptide SIINFEKL derived from OVA or SVYDFFVWL derived from TRP-2 were kindly provided by T. Schumacher (Amsterdam, The Netherlands). Single cell suspensions prepared from spleens were incubated with rat anti-mouse anti-CD8-FITC and anti-CD3-PerCP-Cy5.5 mAbs (both BD Biosciences) together with the respective tetramer for 40 min at 4 °C. Acquisition was performed by flow cytometry using FACSCalibur with CELL-Quest software (BD Biosciences) with dead cells exclusion based on scatter profile or propidium iodide inclusion. FlowJo software was used to analyze at least 300,000 events.

### 4.5. Immunohistochemistry

Tumors, LN, livers and lungs from *ret* transgenic mice were fixed in 4% formalin solution for 24 h at 4 °C followed by embedding into the paraffin. Consecutive cryostat sections 5 μm in thickness were air-dried, treated with xylol and ethanol and stained with polyclonal Abs against TRP-1, TRP-2, gp-100 and tyrosinase as previously described with some modifications [44]. After staining with secondary biotinylated goat anti-rabbit Ab (Vector Laboratories, Burlingame, CA, USA), the sections were treated with Vectastain ABC-AP and Red Alkaline Phosphatase Substrate Kits (both from Vector Laboratories) according to the manufacturer’s protocol and counterstained with hemalaun.

### 4.6. Synthetic Peptides

The H2-K^b^-restrictd T cell epitopes specific for OVA (SIINFEKL) [45] or TRP-2 (SVYDFFVWL) [22,23] together with the IA^b^-restricted T helper epitope (TPPAYRPPNAPIL) derived from HBV core antigen [46] were generated by Fmoc chemistry and were subsequently purified by HPLC at the peptide synthesis core facility of the DKFZ.

### 4.7. IFN-γ ELISPOT Assay

Splenocytes were isolated 12 days after immunization and cultured with 100 ng of respective peptides or 10^4^ EG7 stimulator cells (5 × 10^5^ spleen cells/well) in ELISPOT plates (Nunc, Langenselbold, Germany) coated with 10 μg/mL rat anti-mouse IFN-γ capture mAbs (Biosource/Invitrogen, Karlruhe, Germany). 18 h later, washed plates were incubated with 2 μg/mL biotinylated rat anti-mouse IFN-γ mAbs (BD Bioscience) followed by the treatment with avidin conjugated alkaline phosphatase (BD Bioscience) and 50 μL AEC substrate solution (Sigma, Deisenhofen, Germany). Spots were counted using a Bioreader 3000 (Biosys, Karben, Germany).

### 4.8. ELISA

ELISAs were performed with OptEIA IFN-γ or OptEIA IL-2 ELISA sets (BD Bioscience) according to the manufacturer’s instructions. Briefly, Maxisorp ELISA plates (Nunc, Langenselbold, Germany) coated with capture mAbs were incubated with 100 μL of supernatant per well followed by the treatment with biotinylated detection mAbs together with the streptavidin-conjugated horse reddish peroxidase. 3,3',5,5'-tetramethylbenzidine (TMB) was added as a substrate. The reaction was stopped by adding 1 M H_3_PO_4_. OD_450_ was measured with an ELISA-reader (SLT-Labinstruments, Achterwehr, Germany).

### 4.9. Data Analysis

Student’s *t* test was used to determine statistical significance between control and test groups.

## 5. Conclusions

The *ret* transgenic mouse model of spontaneous melanoma allows investigation of the interactions between tumor and T cells during melanoma progression that closely resembles the clinical situation. Moreover, this preclinical model could be suitable for the development of novel immunotherapeutic approaches to the treatment of human malignant melanoma.
